# Phone addiction, cyberbullying, and mental health amongst young adults in the United Arab Emirates: a cross-sectional study

**DOI:** 10.1186/s40359-023-01320-1

**Published:** 2023-10-06

**Authors:** Nour AlQaderi, Ahmed Banibella Abdelmagied Elamin, Khadiga Yasser Abdelraouf Abdelmonem, Hajar Jamal Teir, Gabriel Andrade

**Affiliations:** Ajman University, Ajman, United Arab Emirates

**Keywords:** Smart phone addiction, Cyberbullying, Anxiety, Depression, Adults, United Arab Emirates

## Abstract

**Background:**

Smartphone addiction is a common phenomenon worldwide and within the UAE. It is related to many factors, including gender and ethnicity, and can lead to mental health disorders, such as anxiety and depression. This study investigates these factors concerning smartphone addiction among young adults in the UAE.

**Methods:**

421 participants answered a questionnaire of validated and reliable scales measuring smartphone addiction, cyberbullying experiences, mental health, and demographic information. The average age of the participants was 21 years, and the age groups were divided into two groups. Group 1 consists of participants who are 20 years or younger, and group 2 consists of participants aged 21 or older.

**Results:**

There was a positive correlation between smartphone addiction with both anxiety and depression. A positive correlation was also found between cyberbullying victims, anxiety, and depression. Females were found to have higher levels of anxiety and smartphone addiction in comparison to males.

**Conclusion:**

Smartphone addiction is a problem that connects to disorders like anxiety and depression. Conversely, cyberbullying is not directly related to smartphone addiction but is also strongly related to anxiety and depression.

**Supplementary Information:**

The online version contains supplementary material available at 10.1186/s40359-023-01320-1.

## Introduction

Since the 21st century is termed as “the age of information technology”, the usage of smartphones has permeated our day-to-day lives and has become virtually ubiquitous [1]. Every day, smartphones are becoming increasingly indispensable to many people’s lives worldwide. It has substantially changed how we work, communicate, and interact with the world around us. Furthermore, it is further substantiated by the fact that in 2020, approximately 61.62% (4.78 billion) of the global population were found to be smartphone users [2].

While smartphones are very efficient and convenient, excessive use of these devices has the potential to lead to smartphone addiction. An expanding amount of recent evidence has implied that smartphone addiction is closely correlated with adverse cognitive and behavioral issues, including but not limited to depression and anxiety. Researchers, therefore term the surge of smartphone usage as a “double-edged sword” [3].

These psychological issues tend to be found more among women than in men. According to widespread research, women tend to encounter more significant levels of anxiety than men. This is further established by research done by the National Comorbidity Survey (1990 to 1992), which illustrated that the lifetime incidence rates for anxiety disorders were 19.2% for men and 30.5% for women [4]. The correlation between depression and anxiety could be due to factors such as perpetual notifications, the pressure to reply to messages, and the fear of missing out on recent news or events. Moreover, another risk factor for anxiety and depression is poor sleep quality. Excessive smartphone use often disturbs sleep processes and can even augment psychological stress, which can negatively affect the sleep cycle [5]. In like manner, social media usage on smartphones contributes to poor self-esteem and mental health, which yet again can exacerbate the symptoms of depression and anxiety [6].

Furthermore, an additional factor that researchers presume to increase depression and anxiety from smartphone use is cyberbullying. Cyberbullying is best described as “willful and repeated harm inflicted through the use of computers, cell phones, and other electronic devices” [7].

A taxonomy on the various types of cyberbullying was created by Willard et al. [8] which includes harassment (i.e., multiple insulting messages sent to a target), flaming (i.e., an online brawl), outing and trickery (i.e., seeking personal information from an individual and sharing it without their consent), impersonation (i.e., personifying the victim and electronically delivering negative information to others as if it were coming from the victim), exclusion (i.e., blocking an individual from their contact list), cyberstalking (i.e., stalking an individual electronically by sending repetitive threatening communications), and sexting (i.e., sharing an individual’s nude pictures without their consent).

With the increasing usage of smartphones, many are at risk of being victims of cyberbullying and cyberbullies simultaneously. This can be seen in an abundance in adolescents where studies have been made to deduce the rationale behind such actions, which included seeking revenge on a colleague in school, that it caters to a more convenient method of bullying and jealousy [9]. The acts of face-to-face bullying in schools are known and condemned by the majority. When that occurs, adolescents might find it suitable to bully another with the anonymity of the internet. As such, more light is needed on the relation of smartphone addiction to cyberbullying. In other parts of the world, it has been well-established that smartphone addiction is positively correlated to being a victim of cyberbullying and depression [10]. But in reality, that could be the opposite for those in the UAE. Although the UAE has produced campaigns against cyberbullying and aided in its awareness, the lack of knowledge on the matter still warrants concern about how smartphone addiction could correlate to bullying.

While the majority of awareness has been given to the adverse effects of being a victim of bullying, research has shown that being a victim can also be a significant risk factor for externalizing problems [11]. These effects can manifest and consequently cause the victim to become a bullying aggressor, otherwise known as a victim bully.

Studies have found a strong correlation between being a victim of bullying and subsequently engaging in bullying behavior or criminal acts in the future [12]. The reasons for this correlation are complex and multifaceted. Being a victim of bullying can include being ridiculed, excluded, and shunned by peers, and physically targeted. Subsequently leading to feelings of anger, isolation, frustration, fearfulness, and helplessness, which can manifest in anger, conduct problems, and aggressive behavior towards others and, at times, even to themselves [13, 14]. Furthermore, individuals who bully and are bullied are at a greater risk of experiencing both externalizing factors and internalizing ones, such as anxiety and depression [3]. A study revealed that adolescents who cyberbullies are inclined to do so to impress their peers and gain a greater social standing [9]. These attributes can further cause a bully to manifest internalized problems as they might fail to achieve the social power they desire. Moreover, some victims of bullying may cultivate an inclination for revenge or seek to gain power and control by becoming a bullying aggressor. Additionally, exposure to being bullied can lead to changes in both cognitive and emotional functioning, including a reduction in empathy and a greater acceptance of violence [15]. These developmental differences can make it easier for individuals who have been victimized to engage in bullying behavior toward others.

Consequently, it is vital to acknowledge that being a victim of bullying can have long-lasting consequences on an individual and can lead to a cycle of aggression and victimization. The means to successfully manage bullying should include effective interventions that should focus on addressing the behavior of bullying aggressors and supporting victims to avert them from becoming aggressors themselves later on.

In this study, we will ask the following research questions: (1) In the UAE, what are the levels of the following variables: phone addiction, cyberbullying, depression, and anxiety? (2) How do these levels vary across gender and ethnicity? (3) Are there any correlations among such variables? The information obtained will be useful for policymakers in the UAE to formulate efficient action plans in order to improve the well-being of the population.

## Methods

### Design and participants

The design of the study was cross-sectional. Information on three demographic variables (age, gender, ethnicity) and four other variables (smartphone addiction, anxiety, depression, cyberbullying victimization, and cyberbullying aggression) would be observed, and various correlations would be established.

Sample size was calculated based on Slovin’s criterion, as typically recommended by statisticians [16]. With an estimated population of 10,000,000 in the UAE, a margin of error of 5%, and a confidence level of 95%, the required sample size would be 385. However, anticipating that the response rate would not be 100%, 450 surveys were sent.

Additionally, a power analysis was done to calculate the minimum sample size in order to avoid type-II errors. For a two-tailed correlation under a bivariate normal model, with a medium effect size (0.3), minimum desired power of 0.8, and α < 0.05, power analysis revealed that the sample should consist of at least 84. For a two-tailed t-test, with medium effect size (Cohen’s d = 0.5), minimum desired power of 0.8, and α < 0.05, power analysis revealed that the sample should consist of at least 128 (64 in each group). These requirements were met for all tests.

Sampling was non-probabilistic; recruitment was done based on availability and willingness to answer the survey. Nevertheless, some level of stratification was used, in order to ensure that the sample included adequate representatives of both genders and ethnicities (Arabs and non-Arabs). Inclusion criteria were having sufficiency in the English language and being a resident of the UAE.

Responses were collected in face-to-face interactions in various venues in the UAE (universities, malls, public spaces, etc.). Participants were shown a QR code, they scanned it, and responses were kept in a depository of Microsoft Forms. Only questionnaires with complete answers were kept. 421 surveys were returned with complete answers, the response rate was, therefore 94%. Participants were given sufficient time to reflect on their responses to ensure the data’s accuracy. Given that the data was entered directly by participants on their own devices (phones, tablets, laptops), the risk of wrongly entering the data was kept at a minimum.

Mean age was 21.1, standard deviation = 4.48, median = 20. Participants were divided into two age groups, using the median as a divider. Participants aged 20 or younger were considered “Age group 1”, participants aged 21 or older were considered “Age group 2.”

### Measures

The survey was composed of four parts. First, demographic information was collected: age, gender, and ethnicity. Gender was obtained by asking participants, “What is your gender?” with “Male/Female” alternatives. Ethnicity was obtained by asking the participants, “Are you an Arab?” with “Yes/No” alternatives.

Second, the Smartphone Addiction Scale (SAS) was included. This is a scale that assesses participants’ levels of phone addiction [17]. Participants are requested to express their agreements on the basis of a Likert scale (1 = strongly disagree; 5 = strongly agree), to 10 statements (e.g., “I will never give up using my smartphone even when my daily life is already greatly affected by it”; “I am constantly checking my smartphone so as not to miss conversations between other people on WhatsApp, Facebook, Instagram, etc.”). Higher scores indicate higher levels of phone addiction. The SAS has been validated in various cultural contexts [17–20], including Arab populations [21]. It is also considered to have good reliability [17].

Third, the Patient Health Questionnaire (PHQ-4) was included. The PHQ-4 is an instrument with 4 statements that assess non-clinical subjects’ mental health along two dimensions: depressive symptoms (“Over the last 2 weeks, how often have you been bothered by little interest or pleasure in doing things?”) and anxiety symptoms (e.g., “Over the last 2 weeks, how often have you been bothered by not being able to stop or control worrying?”). Responses are structured around Likert scales (1 = Not at all; 5 = Nearly every day), with higher scores indicating higher levels of depressive symptoms and anxiety. The PHQ-4 has been validated in previous studies [22], and is considered reliable [23].

Fourth, we included a scale that assesses participants’ experience as victims of cyberbullying. This scale has been designed by Patchin and Hinduja [24], and for purposes of the current study, we label it “Cyberbullying Victim Scale” (CVS). The scale is structured around 9 statements (e.g., “Someone posted mean or hurtful comments about me online”, “Someone threatened to hurt me through a cell phone text message.”) Participants report the frequency with which they encounter these situations, on the basis of a Likert scale (1 = never; 5 = many times). This measure has been validated and tested for reliability in previous applications [25].

Fifth, we included a scale that assesses participants’ experience as aggressors in cyberbullying. This scale has been designed by Patchin and Hinduja [24], and for purposes of the current study, we label it “Cyberbullying Aggression Scale” (CAS). The scale is structured around 9 statements (e.g., “I posted mean or hurtful comments about someone online”, “I threatened to hurt someone through a cell phone text message.”) Participants report the frequency with which they encounter these situations, based on a Likert scale (1 = never; 5 = many times). This measure has been validated and tested for reliability in previous applications [25].

### Data analysis

Data was tested for outliers across variables using Grubb’s test; 1 outlier was identified for age; 1 outlier was identified for CVS; 1 outlier was identified for CAS. Outliers were removed from the data; the remaining sample size was therefore 418.

Independent, two-tailed Student’s t-test analyses were done with gender as the grouping variable, and SAS, the anxiety dimension of PHQ-4, the depression dimension of PHQ-4, CVS, and CAS as dependent variables. As explained by Ghasemi & Zahediasl [26], with large enough sample sizes “we can use parametric procedures even when the data are not normally distributed;” since in this study sample size was sufficiently large, no normality assumptions were needed for t-tests. Homogeneity of variance assumption was congregated for all t-tests (Levene’s test p-values > 0.05). Effect size (Cohen’s d) was also calculated for all analyses. Independent, two-tailed Student’s t-test analyses were done with ethnicity as the grouping variable, and SAS, the anxiety dimension of PHQ-4, the depression dimension of PHQ-4, CVS, and CAS as dependent variables. Since in this study sample size was sufficiently large, no normality assumptions were needed for t-tests. Homogeneity of variance assumption was met for every variable except CAS (Levene’s test p-value < 0.05); consequently, Welch’s test was used for CAS. Effect size (Cohen’s d) was also calculated for all analyses. Independent, two-tailed Student’s t-test analyses were done with age group as the grouping variable, and SAS, the anxiety dimension of PHQ-4, the depression dimension of PHQ-4, CVS, and CAS as dependent variables. Since in this study sample size was sufficiently large, no normality assumptions were needed for t-tests. Homogeneity of variance assumption was met for every variable except Depression (Levene’s test p-value < 0.05); consequently, Welch’s test was used for Depression. Effect size (Cohen’s d) was also calculated for all analyses.

**Fig. 1:**
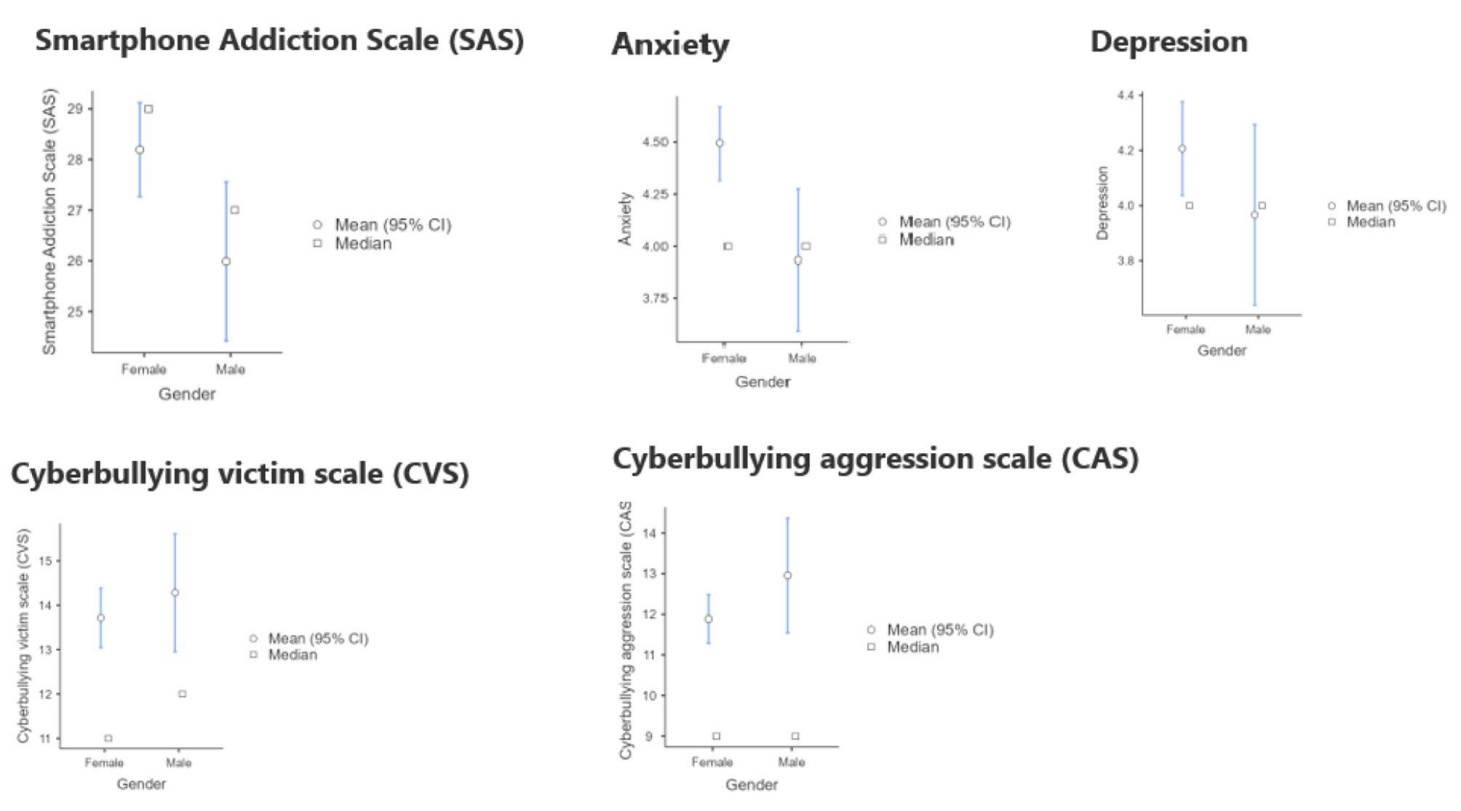
Gender comparisons

Spearman’s coefficient matrix was calculated for the correlation of these variables: SAS, the anxiety dimension of PHQ-4, the depression dimension of PHQ-4, CVS, CAS, and age. The monotonic assumption was verified by graphing the correlations (see Fig. 1). Spearman’s test was used instead of Pearson’s, given that most statisticians recommend using the former instead of the latter, when one (or more) of the variables correspond to a rank level of measurement. Given that the SAS is based on a Likert scale, it relies on a rank level of measurement, and consequently, in this case, Spearman’s coefficient is more adequate [27, 28].

Statistical significance was placed at p < .05. Statistical analyses were done with Jamovi software, a reliable tool that is frequently used by statisticians [29].

## Results

Descriptive results are presented in Table 1. 78% of respondents were females, and 22% were male. 72.2% were Arabs, 27.8% were non-Arabs.

**Table 1:** Descriptive results

	Age	Smartphone Addiction Scale (SAS)	Anxiety	Depression	Cyberbullying Aggression Scale (CAS)	Cyberbullying Victim Scale (CVS)
**N**	418	418	418	418	418	418
**Mean**	21.1	27.7	4.38	4.16	12.1	13.8
**Median**	20	28	4	4	9	11
**Standard deviation**	4.48	8.42	1.65	1.58	5.84	6.24
**Minimum**	16	10	2	2	9	9
**Maximum**	60	50	8	8	41	44

Statistical tests with gender as grouping variable and SAS, the anxiety dimension of PHQ-4, the depression dimension of PHQ-4, CVS and CAS as dependent variables, are presented in Table 2, and the plots are presented in Fig. 1. Gender comparisons can be visually assessed in Fig. 1. Women have statistically significant higher levels in SAS (small effect size) and anxiety (small effect size). There are no statistically significant differences across genders in terms of depression, CVS, and CAS.

**Table 2:** Comparison tests with gender as grouping variable

Variable	Test	Statistic	df	P	Effect size test	Effect Size
Smartphone Addiction Scale (SAS)	Student’s t	2.203	416	0.028	Cohen’s d	0.2632
Anxiety	Student’s t	2.876	416	0.004	Cohen’s d	0.3437
Depression	Student’s t	1.278	416	0.202	Cohen’s d	0.1527
Cyberbullying Victim Scale (CVS)	Student’s t	-0.759	416	0.448	Cohen’s d	-0.0907
Cyberbullying Aggression Scale (CAS)	Student’s t	-1.537	416	0.125	Cohen’s d	-0.1836

**Fig. 2:**
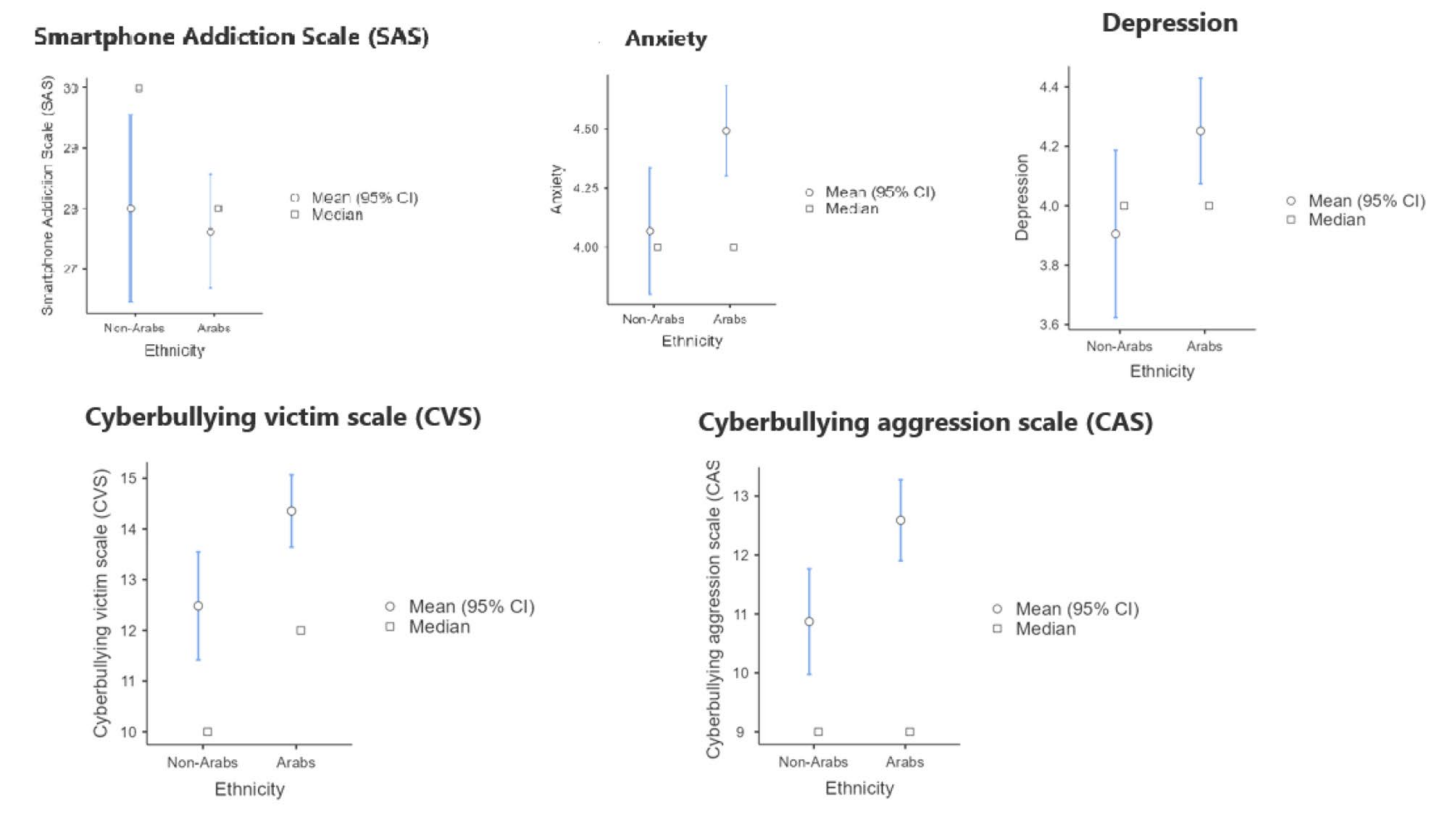
Ethnicity comparisons

Statistical tests with ethnicity as grouping variable and SAS, the anxiety dimension of PHQ-4, the depression dimension of PHQ-4, CVS and CAS as dependent variables, are presented in Table 3, and the plots are presented in Fig. 2. Arabs have statistically significantly higher levels of anxiety (small effect size), depression (small effect size), CVS (small effect size), and CAS (small effect size). There are no statistically significant differences across ethnicity in terms of SAS.

**Table 3:** Comparison tests with ethnicity as a grouping variable

Variable	Test	Statistic	df	P	Effect size test	Effect Size
Smartphone Addiction Scale (SAS)	Student’s t	0.414	416	0.679	Cohen’s d	0.0452
Anxiety	Student’s t	-2.365	416	0.018	Cohen’s d	-0.2583
Depression	Student’s t	-2.021	416	0.044	Cohen’s d	-0.2208
Cyberbullying Victim Scale (CVS)	Student’s t	-2.767	416	0.006	Cohen’s d	-0.3022
Cyberbullying Aggression Scale (CAS)	Welch’s test	-2.715	416	0.003	Cohen’s d	-0.3102

**Fig. 3:**
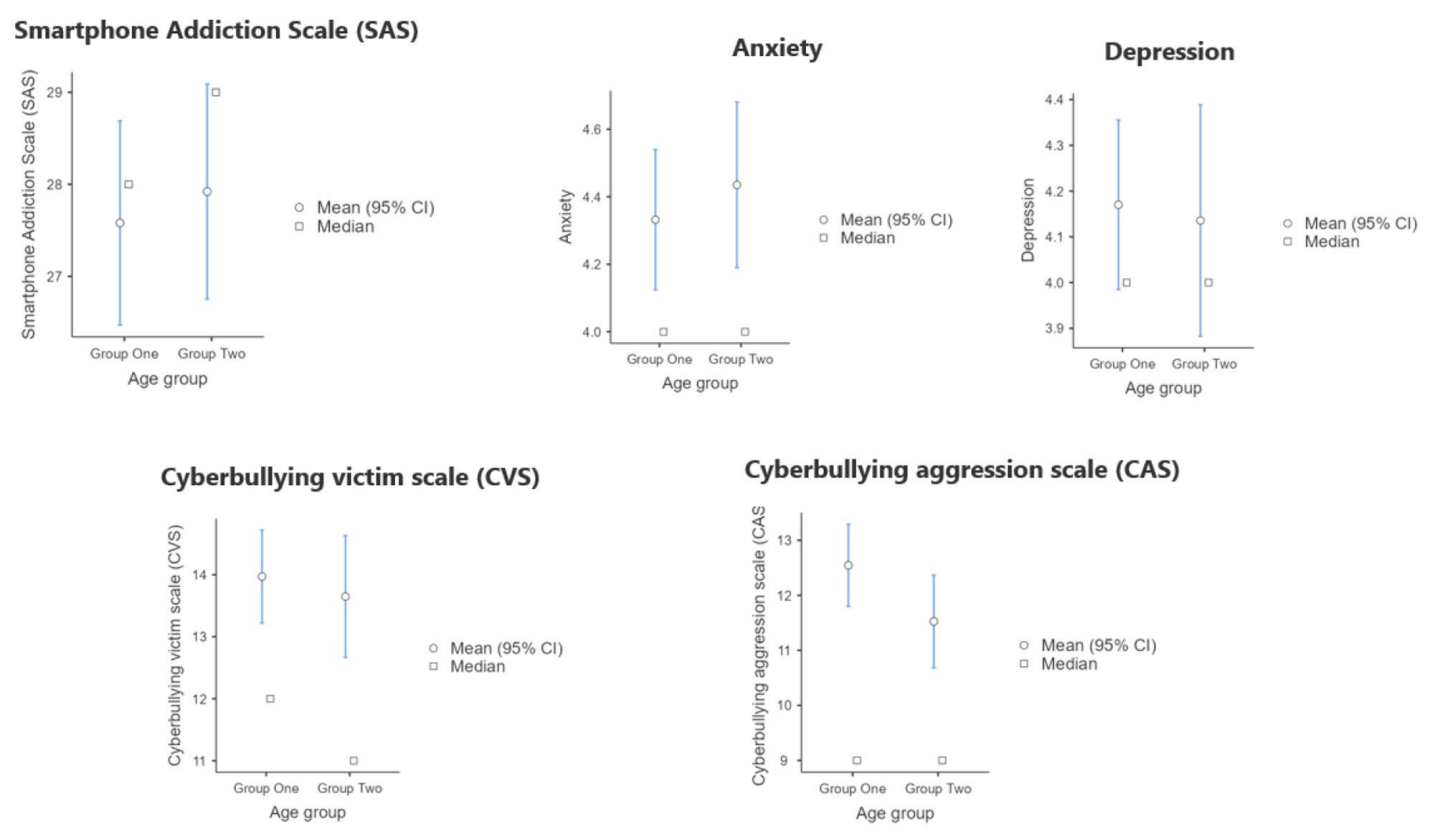
Age group comparisons

Statistical tests with age group as grouping variable and SAS, the anxiety dimension of PHQ-4, the depression dimension of PHQ-4, CVS and CAS as dependent variables, are presented in Table 4, and the plots are presented in Fig. 3. There are no statistically significant differences across age groups in any of the variables.

**Table 4:** Comparison tests with age group as a grouping variable

Variable	Test	Statistic	df	P	Effect size test	Effect Size
Smartphone Addiction Scale (SAS)	Student’s t	-0.408	416	0.684	Cohen’s d	-0.04
Anxiety	Student’s t	-0.63	416	0.53	Cohen’s d	-0.06
Depression	Welch’s test	0.22	343	0.83	Cohen’s d	0.02
Cyberbullying Victim Scale (CVS)	Student’s t	0.52	416	0.60	Cohen’s d	0.05
Cyberbullying Aggression Scale (CAS)	Student’s t	1.77	416	0.07	Cohen’s d	0.17

Matrix of Spearman’s correlations for all numerical variables (SAS, the anxiety dimension of PHQ-4, the depression dimension of PHQ-4, CVS, CAS, and age) is presented in Table 4. There are statistically significant positive correlations between SAS and anxiety (medium effect size), SAS and depression (medium effect size), depression and anxiety (large effect size), anxiety and CVS (small effect size), depression and CVS (small effect size), and CVS and CAS (large effect size). There is a statistically significant negative correlation between SAS and CAS (small effect size).

## Discussion

### General patterns

Excessive smartphone usage is a problem that the Middle East and North Africa (MENA) region, as well as other regions of the world, suffer from. A cross-cultural study conducted in four countries within the MENA region illustrated the varying prevalence of problematic and excessive smartphone use: 59.8% in Jordan, 27.2% in the Kingdom of Saudi Arabia (KSA), 17.3% in Sudan, and 8.6% in Yemen [30]. Other regions of the world also had fluctuating figures ranging from 38.9% in the United Kingdom, 30% in Malaysia, and 12.5% in Spain [31–33]. As for age our results show no statistical significance between age groups across variables, as demonstrated in Table 4. This result is quite surprising as many studies indicate that cyberbullying tends to peak during adolescence and then steadily declines [34]. Studies regarding cyberbullying remains scarce within the MENA region, so this finding could be a phenomenon experienced just within this region. However, other studies conducted in Western countries produced varying results. In New Zealand, a national study overlooking cyberbullying victimization across gender, age, and ethnic differences involved over 20,000 participants between 18 and 97. Moreover, 14.9% responded as being victims of cyberbullying, young adults (18–25) were reported to be the largest demographic at target, while prevalence was lowest with the older adults (66+) [35]. Meanwhile, another study conducted in the United States of America had participants ranging from elementary students to university students, with the principle focus being on age and gender, found that most cyberbullying victims and perpetrators were middle schoolers, spanning grades 6 to 8. Highschool and university students depicted a decreased participation in cyber bullying, while elementary students had the least involvement. Over the course of the study, gender did not play a relevant part [36].

### Gender differences

Within the UAE, the results shown in Table 2 reveal that there are significant gender differences amongst the levels of smartphone usage, with women having significantly higher levels of smartphone usage in comparison to men, as it appears to be a common finding in studies within the MENA region and other parts of the world. For instance, a multicenter study in the KSA highlighted that female university students were more addicted to their smartphones than male university students [37]. In like manner, another study reported that South Korean female adults tend to excessively use their smartphones compared to males [3].

There are many theories on why women might be susceptible to excessive smartphone use. Firstly, various studies reveal that women value social interactions more than their male counterparts. Women are more sensitive to the oxytocin secreted when interacting with people socially, hence why women tend to have the feeling of being more rewarded and satisfied [38]. Due to this, women have an increased likelihood of using their smartphones for social interaction. Furthermore, it is achieved through the use of smartphones to stay in touch with relatives and friends via text, voice and video calls, or other social media applications. They were resulting in dependency on their smartphone devices to reap this gratifying feeling. Furthermore, this addiction does not spare the younger population of girls. In a recent study conducted in Sweden, primary and secondary school female students have been shown to have excessive smartphone usage of 60%, whereas male students have 30% [39].

Moreover, in developing countries, the ownership and usage of smartphones is almost vital to the emancipation of females. The plethora of benefits that women reap from smartphones include, but are not limited to, financial independence, employment, better family health, and education [40]. Although excessive smartphone use is a problem for both genders, further research needs to be done to explore the underlying cause of this gender discrepancy in smartphone usage.

The results in Table 2 also depicted that anxiety was quite higher in females than in males within the UAE. This finding is not surprising at all as countless studies (including countries within the MENA region, like the UAE, KSA, and Kuwait) have illustrated this finding repeatedly [41–45]. Many researchers have a plethora of explanations as to why there is a gender difference between anxiety levels. The first explanation could simply be due to the different biological makeup of males and females. Females have rhythmic changes in gonadal hormones along the stages of their lives, from puberty and pregnancy to menopause. Throughout their lives, females’ brains are constantly exposed to fluctuating hormone levels, which can actively impact their cerebral function and neurochemistry and contribute to neuropsychiatric conditions like anxiety disorders [46].

Secondly, another explanation is primarily due to current cultural and societal influences. This explanation was further endorsed by a recent study conducted in the UAE, which highlighted how cultural and lifestyle factors are causing a significantly higher rate of anxiety in female adolescents in comparison to male adolescents [47]. This is mainly because women today face significant societal pressure to conform to the norms, whether concerning their beauty, careers, or families. This constant external pressure that they face tends to trigger feelings of being overwhelmed, low self-esteem, and apprehension. Consequently, these feelings inadvertently can further start women to have a higher propensity for anxiety. The primary source of these societal and cultural pressures is most commonly exhibited through social media use [48]. Therefore, the conclusion can be made that women who use their smartphones excessively are more prone to have feelings of anxiety [49]. As mentioned previously, women tend to have higher dependency and attachment to their smartphones and due to this, Annoni et al. found that women reported elevated levels of anxiety when their smartphones were not within reach and felt a general attachment to their smartphones.

According to the studies mentioned above, it is clear that women are more predisposed to anxiety than men, which emphasizes the importance of identifying these potential risk factors to improve the lives and mental health of women not only in the UAE but worldwide. This can be done by enforcing early preventative measures and trying to reduce societal and cultural pressures with the help of social initiatives within the UAE and by utilizing different national social media platforms to help raise awareness.

Contrarily, this study showed that there were no significant differences across genders concerning depression and becoming a cyberbullying aggressor or victim. In terms of the results for depression, this is quite a surprising finding as one would expect the levels of depression to be increased in females, alongside the increased levels of anxiety. This assumption is further substantiated by many studies, which show that women experience depression and anxiety at considerably higher rates than men [50, 51]. In a study in Al Ain, UAE, higher lifetime rates of depression were also reported in females than in males [52]. This finding of no gender differences in the rate of depression in this study can be related to the fact that feelings of depression often manifest differently in both men and women, which in turn might make it difficult to recognize [53]. Therefore, we must raise awareness that depression can affect anyone, no matter their gender, and there should be a promotion of a higher scope of awareness of the different ways depression can manifest. This can be done by establishing general campaigns to increase mindfulness of mental health issues in UAE-based workplaces and educational institutions. Likewise, launching initiatives in the UAE to reduce the stigma associated with mental illness and broaden access to various types of mental health care is crucial.

In like manner, there were no gender differences found with becoming a cyberbullying aggressor and victim. Numerous research studies have yielded inconclusive results regarding the role of gender. Therefore, whether or not these differences in becoming a victim or aggressor to cyberbullying amongst genders remains ambiguous [54]. Regardless of gender differences, cyberbullying can affect anyone within the population. Promoting a polite and safe online environment is the first action in combatting cyberbullying within any society, and it is currently being implemented within the UAE, where according to the severity of the offense, penalties or fines can be imposed as cyberbullying is considered a serious offense [55]. However, further awareness programs and preventative initiatives within the UAE could further ameliorate cyberbullying.

### Ethnicity comparison

Findings from our study have indicated a significant relationship between Arabs and traits of anxiety with depression in Table 3. It is commonly known that mental health is disregarded in the Arab world, even though many of its inhabitants are exposed to conflicts, wars, and terrorism, which leads to numerous behavioral and mental disorders. Cultural beliefs about the evil eye and the impact of possessions produce an altered interpretation of cognitive symptoms as more concerning data from Okasha et al. [56] showcases that most individuals seek aid from traditional healers than today’s physicians; this may be due to the importance placed by Arab society on traditional healers, as they claim to deal with supernatural phenomena. The current dilemma is noted to be found in all the 22 members of the Arab League (Algeria, Bahrain, Comoros, Djibouti, Egypt, Iraq, Jordan, Kuwait, Lebanon, Libya, Mauritania, Morocco, Qatar, Oman, Palestine, Saudi Arabia, Somalia, Sudan, Syria, United Arab Emirates, Tunisia, and Yemen), which accounts for over 425 million individuals.

National studies conducted by Morocco and Egypt illustrated significant results on mental disorders across both nations. Morocco reported a point prevalence of 26.5% for major depressive disorder and 9.3% for generalized anxiety disorder. The Egyptian study showcased a point prevalence of mood disorder of 6.4% and 4.8% for anxiety disorder [57, 58]. As a result of the stigma, unwillingness to self-disclose, and lack of usage of legal services to aid their illness [59].

As such, it could be subdivided into two categories: health care setting & socio-cultural level [60]. In healthcare settings, stigmatization persists among Arabs as they see care for mental illness as too specialized for it to be part of primary care [61] Furthermore, there is a shortage of psychiatrists in the region as medical students lack interest in pursuing such a career following the local stigmatization in the Arab community, which is worsened by the scarcity of mental health nurses, healthcare settings, and funding in Arab communities. In a study done in KSA, psychiatric patients felt more at ease with physicians than psychiatrists due to the local stigmatization [61]. In addition, people residing in Oman reported a preference for facilities for psychiatric care to be placed away from the community [62].

The socio-cultural level could be due to a family paradox amongst Arabs. The family’s identity in their community could be ruined as a deeply rooted fear can break families apart [63]. Mainly due to “stigma by association” as it could destroy the family’s business and prospect of marriage and increase the chances of divorce [64]. This is entailed by social shame, not only for the patient but for their family as well.

In our study, it was noted in Table 3 that there was no significance in terms of ethnicity and its relation to SAS, despite it going against the common belief that Arabs are at a high risk of phone addiction compared to their counterparts in Europe and the Far East. A study was conducted in the UAE to identify problematic phone usage among college-aged adults [65]. Results report a prevalence of problematic phone usage in 29% of the sample population. A similar study was also conducted in Lebanon that reported an inappropriate phone usage of up to 20% in a sample population older than previously mentioned [66]. In comparison, those in Europe and the Far East are far better off than Arabs, whose problematic phone usage was from 1 to 15% [33, 37, 67].

Regarding being a cyberbully victim or aggressor, Arabs as an ethnic group were found to have higher levels than non-Arabs. Studies conducted in Saudi Arabia indicated the prevalence of cyberbullying as 31.5% (2008) and 29.6% (2013) in 2 separate studies [68]. Furthermore, a study in Saudi Arabia, including 1012 university students, revealed that over half acknowledged being harassed, bullied, or stalked online [69]. Another study by Alrajeh et al. (2021) involving 836 university students in Qatar revealed a concerning percentage of 35.8% being cyberbullying victims, whilst 6.8% were considered cyberbullies [70].

Several studies have been produced about cyberbullying in Latin America due to the growing interest across the continent. An example is Chile, which has a prevalence of 11.4% cyberbullying victims compared to the 12.5% cyberbullying aggressors, with majority in Argentina and Colombia reporting an alternative reality. Those results demonstrate a higher prevalence of cyberbullying aggression in their population than being victims [71].

With such results, more diligent work is needed to identify the reasons which place Arabs in such dilemmas, despite not having any correlation of such factor on their smartphone addiction to their ethnicity.

### Correlation of smartphone addiction with cyberbullying and mental health

As previously mentioned, smartphone addiction is a prevalent concern in present times that can have a substantial impact on one’s mental health and the likelihood of being a cyberbully. Table 4 of this study shows a positive correlation between smartphone addiction and anxiety. This can be further augmented by research done in Korea, where 1733 adolescents partook in a study that showed that in conjunction with increased usage of smartphones, there was an increase in anxiety, which consequently led to worsening physical and psychological health [72]. The increase in anxiety can be correlated with a diminished time for sleep as people who extensively use their phones would postpone their sleeping time and do so when a person should rest. A two-wave cohort study that included 4,175 youths stated that sleep deprivation of 6 h or less in a night exacerbated major depression [73]. In relation, a study conducted in 2015 with adolescents as the primary participants revealed that smartphone addiction also caused depression alongside anxiety, which can be the result of a decline in sleeping hours due to a diminished potential towards self-control [74]. This conclusion can also be seen in another study conducted that concluded that the increase in social media consumption can cause depression in children, adolescents, and young adults because of decreased sleep when compared to the usage of social media in a decreased amount [75].

Adjacent to the positive correlation of anxiety with smartphone usage, there is also a positive connection with depression as proven in an analysis done in Egypt where 1,380 undergraduate Egyptian students showcased a relationship in great magnitude regarding both mental health disorders [56]. In some cases, they go hand in hand, which seems to be the case in the present study where a significant correlation was found between anxiety and depression. As an individual uses their smartphone more in an uncontrolled manner, a greater capacity for depression becomes feasible. Analogous to this is a study done across the MENA region where problematic smartphone usage was heavily associated with both anxiety and depression, as well as stress [41]. A study conducted amongst university students in Lebanon found that there was a statistically significant correlation between an individual with depression and anxiety, and smartphone usage, where it was deduced that its addiction could be due to a need for a coping mechanism or is used as an approach to managing one’s mood [76]. In this study, it is proven that not only are anxiety and depression are linked to each other and display a strong manifestation in their connection to being a victim of cyberbullying.

It is deducible that the mental health of someone who is a victim of cyberbullying can manifest negatively. The type of aggression projected through a screen can be done in various ways that can cause humiliation, sadness, anger, isolation, and guilt. These emotions can manifest into more significant concerns, such as anxiety and depression. A study amongst university students showcased that 238 out of 1,044 victims of cyberbullying during two previous months reported increasingly high levels of stress, depression, and anxiety. Consequently, more than 60% of the students ultimately disclosed suicidal ideation due to their cyber victimization [77]. Another study, which further supports the notion that cyberbullying can cause depression, was conducted where a correlation was found between them that suggested the victims experienced mild to moderate depression [75].

Additionally, a retrospective study was conducted in both a Spanish university and a Bolivian university, where 1,593 students were asked to recall whether they were victims of cyberbullying in high school. Amongst the students who endured the bullying, they indicated higher levels of anxiety and depression in comparison to the students who were either bystanders or who had not suffered from cyberbullying; consequently, this precipitated into a further predicament where it was revealed that adjusting psychologically to university was formidably more challenging [78]. The occurrence of falling victim to cyberbullying leading to anxiety and depression can also be regarded in a cross-sectional study conducted in private schools in Lebanon, where both variables were consistently seen when malicious and demeaning content was directed at the students [79]. Another study stated that when someone finds themselves addicted to something, depression usually follows, as they both go hand in hand. This study revealed that following a four-year study, it was confirmed that addiction to mobile phones, and subsequently to the internet, is a substantial cause of depression and, in the long run, can cause an increased probability of cyberbullying, especially in high school students [10].

In the concept of being a cyberbullying aggressor, within this study, there was a negative correlation related to smartphone addiction. With all the emotions that come with being a bullying victim, a contradictory effect occurs, where the victim also becomes the bully.

In comparison to traditional bullying, where the bully and victim are physically in each other’s presence, cyberbullying has become a rapidly increasing sight in the modern world of technology. This study found a strong correlation between being a victim of cyberbullying and becoming a cyberbully. As previously mentioned, being a victim can cause several negative and internalizing emotions, such as depression and anxiety. Still, they can also cause externalizing symptoms, including aggression, anger, and resentment, manifesting as conduct issues or impulsive actions. These individuals go through psychological conditions that can be depicted as a combination of what a bully and a victim experience. Some studies concluded that the transition to becoming a bully-victim could be due to the need to be accepted by peers and people around them, as they feel less integrated with friendships as either a bully or a victim; however, additional studies are needed to ultimately acquire the absolute interpretation [80].

### Limitations

Although this study’s sample surpassed the minimum size required by both Slovin’s criterion and power analysis, further studies should include larger samples to arrive at more robust conclusions. Likewise, although some stratified criterion was used to ensure the participation of both genders and ethnic groups (Arabs and non-Arabs), reliance on convenient sampling was a limitation. Furthermore, given that women are more likely to respond to surveys [81], there was some gender imbalance in the sample’s composition (with a more significant number of women), thus limiting the extent of the conclusions.

## Conclusion

Mental health becomes a focus when addiction is involved, where it becomes evident that women have higher phone addiction and anxiety levels. Ethnicity is a crucial factor as UAE is a multicultural community, with Arabs having higher levels of anxiety and depression, being cyberbullying victims and aggressors, compared to non-Arabs. Anxiety and depression were also shown to be higher in individuals with smartphone addiction and cyberbullying; therefore, the public authorities need to raise awareness about the importance of using smartphones in a manageable and organized manner to reduce the harmful effects of addiction.

Spearman’s coefficient matrix

**Table:** 

		Age	Smartphone Addiction Scale (SAS)	Anxiety	Depression	Cyberbullying Victim Scale (CVS)	Cyberbullying Aggression Scale (CAS)
**Age**	Spearman’s rho	—					
	p-value	—					
**Smartphone Addiction Scale (SAS)**	Spearman’s rho	0.008	—				
	p-value	0.875	—				
**Anxiety**	Spearman’s rho	0.013	0.311***	—			
	p-value	0.798	< 0.001	—			
**Depression**	Spearman’s rho	-0.064	0.322***	0.632***	—		
	p-value	0.192	< 0.001	< 0.001	—		
**Cyberbullying Victim Scale (CVS)**	Spearman’s rho	-0.019	0.055	0.247***	0.253***	—	
	p-value	0.699	0.264	< 0.001	< 0.001	—	
**Cyberbullying Aggression Scale (CAS)**	Spearman’s rho	-0.091	-0.125	0.029	0.061	0.637***	—
	p-value	0.064	0.011	0.554	0.216	< 0.001	—

*Note.* * p < .05, ** p < .01, *** p < .001

### Electronic supplementary material

Below is the link to the electronic supplementary material.

Supplementary Material 1

## Data Availability

The data of this study is available from the corresponding author (Gabriel Andrade) upon reasonable request.
