# Supplementary material for: Phone addiction, cyberbullying, and mental health amongst young adults in the United Arab Emirates: a cross-sectional study
Source: BMC Psychol. 2023 Oct 6;11:313. doi: 10.1186/s40359-023-01320-1 (PMC10557357; doi:10.1186/s40359-023-01320-1)
Supplement: Supplementary file 1 — Supplementary Material 1 [file 40359_2023_1320_MOESM1_ESM.docx]

**APPENDIX 1**

**Smart phone addiction and cyberbullying**

1.This survey takes about 3 minutes to complete. Your answers are anonymous. You can refuse to answer at any time. Please click below to agree to continue.

Yes

2.What is your gender?

Male

Female

3.How old are you?

Enter your answer

4.Are you Arab?

Yes

No

5.**Please rate your agreement with these statements**  .

|  | Strongly disagree | Disagree | Neutral | Agree | Strongly agree |
| --- | --- | --- | --- | --- | --- |
| I miss planned work due to smartphone use |  |  |  |  |  |
| I have a hard time concentrating in class, while doing assignments, or while working due to smartphone use |  |  |  |  |  |
| I feel pain in the wrists or at the back of the neck while using a smartphone |  |  |  |  |  |
| I won't be able to stand not having a smartphone |  |  |  |  |  |
| I feel impatient and fretful when I am not holding my smartphone |  |  |  |  |  |
| I have my smartphone on my mind even when I am not using it |  |  |  |  |  |
| I will never give up using my smartphone even when my daily life is already greatly affected by it |  |  |  |  |  |
| I am constantly checking my smartphone so as not to miss conversations between other people on WhatsApp, Facebook, Instagram etc. |  |  |  |  |  |
| I use my smartphone longer than I had intended |  |  |  |  |  |
| The people around me tell me that I use my smartphone too much |  |  |  |  |  |

6.**Please express how often you encounter these situations**

|  | Not at all | Several days | More than half the days | Nearly everyday |
| --- | --- | --- | --- | --- |
| Over the last 2 weeks, how often have you been bothered by feeling nervous, anxious or on edge? |  |  |  |  |
| Over the last 2 weeks, how often have you been bothered by not being able to stop or control worrying? |  |  |  |  |
| Over the last 2 weeks, how often have you been bothered by little interest or pleasure in doing things? |  |  |  |  |
| Over the last 2 weeks, how often have you been bothered by feeling down, depressed, or hopeless? |  |  |  |  |

7.**Please express how often you have encountered these situations**

|  | Never | Once | A few times | Several times | Many times |
| --- | --- | --- | --- | --- | --- |
| I have been cyberbullied |  |  |  |  |  |
| Someone posted mean or hurtful comments about me online. |  |  |  |  |  |
| Someone posted a mean or hurtful picture of me online. |  |  |  |  |  |
| Someone posted a mean or hurtful video of me online. |  |  |  |  |  |
| Someone created a mean or hurtful web page about me. |  |  |  |  |  |
| Someone spread rumors about me online. |  |  |  |  |  |
| Someone threatened to hurt me through a cell phone text message. |  |  |  |  |  |
| Someone threatened to hurt me online. |  |  |  |  |  |
| Someone pretended to be me online and acted in a way that was mean or hurtful.  8. **Please express how often you have encountered these situations** |  |  |  |  |  |

|  | Never | Once | A few times | Several times | Many times |
| --- | --- | --- | --- | --- | --- |
| I cyberbullied others. |  |  |  |  |  |
| I posted mean or hurtful comments about someone online. |  |  |  |  |  |
| I posted a mean or hurtful picture online of someone. |  |  |  |  |  |
| I posted a mean or hurtful video online of someone. |  |  |  |  |  |
| I spread rumors about someone online. |  |  |  |  |  |
| I threatened to hurt someone online. |  |  |  |  |  |
| I threatened to hurt someone through a cell phone text message. |  |  |  |  |  |
| I created a mean or hurtful web page about someone. |  |  |  |  |  |
| I pretended to be someone else online and acted in a way that was mean or hurtful to them. |  |  |  |  |  |
